# FVIII Immunogenicity—Bioinformatic Approaches to Evaluate Inhibitor Risk in Non-severe Hemophilia A

**DOI:** 10.3389/fimmu.2020.01498

**Published:** 2020-07-28

**Authors:** Daniel P. Hart

**Affiliations:** Department of Immunobiology, Barts and The London School of Medicine and Dentistry, Blizard Institute, Queen Mary University of London, London, United Kingdom

**Keywords:** inhibitor, *in silico*, risk, non-severe hemophilia, prediction

## Abstract

The life-long inhibitor risk in non-severe hemophilia A has been an important clinical and research focus in recent years. Non-severe hemophilia A is most commonly caused by point mutation, missense *F8* genotypes, of which over 500 variants are described. The immunogenic potential of just a single amino acid change within a complex 2,332 amino acid protein is an important reminder of the challenges of protein replacement therapies in diverse, global populations. Although some *F8* genotypes have been identified as “high risk” mutations in non-severe hemophilia A (e.g., R593C), this is likely, in part at least, a reporting bias and oversimplification of the underlying immunological mechanism. Bioinformatic approaches offer a strategy to dissect the contribution of *F8* genotype in the context of the wider HLA diversity through which antigenic peptides will necessarily be presented. Extensive modeling of all permutations of FVIII-derived fifteen-mer peptides straddling all reported *F8* genotype positions demonstrate the likely heterogeneity of peptide binding affinity to different HLA II grooves. For the majority of *F8* genotypes it is evident that inhibitor risk prediction is dependent on the combination of *F8* genotype and available HLA II. Only a minority of FVIII-derived peptides are predicted to bind to all candidate HLA molecules. *In silico* predictions still over call the risk of inhibitor occurrence, suggestive of mechanisms of “protection” against clinically meaningful inhibitor events. The structural homology between FVIII and FV provides an attractive mechanism by which some *F8* genotypes may be afforded co-incidental tolerance through homology of FV and FVIII primary amino sequence. *In silico* strategies enable the extension of this hypothesis to analyse the extent to which co-incidental cross-matching exists between FVIII-derived primary peptide sequences and any other protein in the entire human proteome and thus potential central tolerance. This review of complimentary *in vitro, in silico*, and clinical epidemiology data documents incremental insights into immunological mechanism of inhibitor occurrence in non-severe hemophilia A over the last decade. However, complex questions remain about antigenic processing and presentation to truly understand and predict an individual person with hemophilia risk of inhibitor occurrence.

## Introduction

The European Union defines a rare disease as one affecting fewer than 5 in 10,000 of the general population, estimating as many as 1 in 17 people will be affected by a rare disease at some point in their lives (1). Hemophilia A is arguably the most well-known and characterized heritable rare diseases. As an X-linked recessive defect in the *F8* gene, the resultant deficiency in FVIII coagulation protein activity (FVIII:C) leads to a phenotype of life long bleed risk. It has been well-established since the 1950s that the severity of this phenotype is inversely correlated to the residual FVIII:C detectable in the person with hemophilia (PWH) plasma (2). Hemophilia A was subsequently classified by the International Society of Thrombosis and Hemostasis (ISTH) as severe, moderate or mild depending on residual measurable FVIII:C, <1, 1–5, or >5 iu/dl, respectively (3). Like some other rare protein deficiency syndromes (e.g., Pompe's disease), therapeutic intervention to moderate the disease phenotype emerged in the form of pre-emptive replacement of the missing protein, so called “prophylaxis.” For severe hemophilia A, prophylaxis was initially in the form of plasma or plasma derivatives (i.e., cryoprecipitate) (4, 5) and subsequent factor concentrates of either donor derived plasma or recombinantly synthesized (6). The predictable immunological consequence of such a protein replacement intervention in a heritable deficiency is one of anti-drug antibodies (ADA) directed against the therapeutic molecule. For PWH, an anti-therapeutic FVIII (t-FVIII) ADA is known as an inhibitor. Inhibitors arising in the early stages of treatment of severe hemophilia A have been well-recognized for as long as the attempts to correct the coagulation protein deficiency (7, 8). Inhibitors are detected using a functional clotting assay (Bethesda assay) and result in partial or complete loss of efficacy of the replacement FVIII therapy depending on inhibitor potency.

Inhibitor occurrence in severe HA is immediately impactful on clinical decision making, necessitating thought about re-establishing tolerance to the FVIII molecule. This “tolerizing” clinical intervention, immune tolerance induction (ITI), is a significant commitment for all concerned: the PWH (most commonly a young boy under the age of 3 years); his parents, hospital treating team and the health service bearing the cost (9, 10). The epidemiology of inhibitor occurrence in the severe HA cohort is now well-described. By the functional, clotting-based surveillance (Bethesda) assay criteria, up to 40% of previously untreated patients (PUPs) will generate a detectable inhibitor. Between 30 and 50% of these will be low titer (<5 Bethesda Units, BU), the remaining majority being much more challenging as high titer (>5 BU) resulting in immediate inactivation of infused t-FVIII concentrate (11, 12). The degree of inherited disruption of the *F8* gene correlates directly with risk for inhibitor occurrence, the more truncated any residual protein product, the higher the inhibitor risk (13). Additional immune response polymorphisms (IRPs) (e.g., IL10, TNF) and intracellular signaling molecules (e.g., MAPK9) have been identified as additional heritable risks for inhibitor occurrence, modified by the environmental influences of treatment exposure intensity and possible FVIII product choice (12, 14–16).

Alongside the considerable work to understand relevance and contribution of IRPs in the generation of inhibitory and non-inhibitory anti-FVIII antibody responses, classification of the immunoglobulin type and subtypes identified class-switching to IgG4 from IgG1 as a predictive step toward a clinically relevant inhibitory ADA (17). Such class switching requires T cell help (Th) and as such tFVIII-derived peptide presentation through HLA class II molecules. Paradoxically, in the context of severe HA, HLA II type seemed to be only a weak determinant of inhibitor risk, likely explicable by the large FVIII protein size providing sufficiently numerous and varied binding peptide sequences for the HLAII repertoire, excluding the likelihood of any allele being predictive. Thereafter, further work to dissect this antigen presentation pathway to understand the key immunological event for inhibitor occurrence in severe hemophilia A declined (18–20).

Although less prevalent in the non-severe HA cohort, and consequently less studied, inhibitor occurrence remains a clinical challenge. Data published from the INSIGHT group has importantly recognized the life-long risk of inhibitor development in this non-severe HA cohort, and that once present, the morbidity and mortality risk is considerable (21, 22). Inhibitory antibodies are detected with the same Bethesda assay as severe hemophilia, although due to the more sporadic, “on-demand” requirement for t-FVIII replacement in moderate and mild HA (non-severe HA) at times of injury or surgery, inhibitory surveillance is not as systematic as severe HA (23). Consequently, the range of 5–13% prevalence of inhibitory activity reported by the Bethesda assay surveillance in non-severe HA may be under reported, but this figure conforms to the observation of a less disruptive *F8* genotype having a reduced risk compared to the larger deletions causing severe HA. Historical collection of *F8* genotype data (e.g., www.f8-db.eahad.org/) has identified >800 missense *F8* mutations resulting in a non-severe HA and whether associated with inhibitor formation. In the last decade it has become very attractive to consider these missense *F8* mutations as an alloreactivity model simplified to the single amino acid difference between the PWH's endogenous FVIII (e-FVIII) and the t-FVIII that risks the anti-drug antibody response and potential inhibitory activity. This review will describe the initial cellular work confirming the value of such a simplified allo-response model to dissect the antigen presentation and T cell activation pathway and subsequent necessity to harness bioinformatic power to explore scaled up hypotheses not amenable to *in vitro* techniques alone.

## Cellular Level T Cell Specific Fviii Peptide Recognition: *in vitro* Work

In 2003, Jacquemin et al. were able to discriminate the helper T cell specificity toward t-FVIII derived peptides containing the position of the wild type Arg2150 FVIII whilst the patient's endogenous His2150 containing peptides were not recognized (24). This correlated with the clinical observation that the R2150H subject of study living with non-severe hemophilia A had an inhibitory response selective for the infused t-FVIII. The elegant experimental design focused on the individual patient's sample with a documented high titer 305BU inhibitor. His PBMC were immortalized, autologous dendritic cells derived to then detect FVIII-specific reactive T cells with IFN gamma secretion read out. Subsequent cloning of reactive CD4 T cells enabled dissection of individual responses in HLA-DR binding assays to confirm differential recognition of R2150 and H2150 containing peptides when presented by DRB1^*^0401/DRB4^*^01 and DRB1^*^1501/DRB5^*^01 HLA Class II haplotypes. These peptide competition assays utilized the concentration of competitor peptide to prevent binding of 50% of biotinylated peptide of interest (IC50). They were also able to abrogate the T cell response by co-culture with a monoclonal antibody to MHC class II DR molecules, indicating the class II restriction being DR specific.

Subsequently, James et al. characterized T-cell responses in two unrelated hemophilia A inhibitor subjects with a different *F8* missense mutation, R593C (25). In contrast to the Jacquemin subject, these 2 subjects demonstrated cross reactivity to their endogenous FVIII sequence, seen clinically in at least 50% of cases of non-severe hemophilia inhibitors (22). Similarly elegant but labor intensive *in vitro* techniques demonstrated the 2 subjects with high titer inhibitors both had HLA DR restricted T cell responses to peptides containing the mutational position 593 in contrast to HLA-DR matched healthy controls. This study's experimental design incorporated some computational biological prediction of peptide binding scores generated by the Propred algorithm alongside conventional competition assays to determine FVIII peptides' affinities to a panel of HLA-DR monomers. Earlier work from the same group had examined Th cell lineage evolution between two brothers, both multiply transfused with FVIII concentrate. Their causative *F8* genotype, A2201P, was different to the aforementioned cases. The proband inhibitor case had a high titer (29BU) inhibitor and a responsive Th2 polarized clone after an earlier Th17/Th1 response, whereas his brother, sharing the HLA-DRA-DRB1^*^0101 allele, without an inhibitor, had detectable but persistently unchanged Th1 clones responsive to *F8* mutation position containing peptides (26).

Taken together these key studies spanning a decade of work, had elegantly dissected the T cell responses of a handful of patients with 3 different *F8* genotypes and <10 HLA-DR alleles (24–26). Labor intensive but informative at the subjects' *F8* genotype and HLA-DR allelic level they addressed key issues of T cell epitope specific allo responses, previously lacking in the severe HA literature. However, they were also emblematic of the future challenges to scale up *in vitro* strategies to address the hundreds of *F8* missense genotypes in the context of more heterogenous HLA Class II presentation. This would be necessary to further understand generalizable mechanisms of inhibitor generation, and to potentially risk stratify inhibitor risk. The key question in both severe and non-severe hemophilia A is not necessarily why an individual has generated an inhibitory response against t-FVIII, but possibly more interesting, why has an individual not generated a clinically meaningful inhibitory response. The emergence of computational biological predictive algorithms offered the potential to model this complexity *in silico* at scale.

## *In silico* Proof of Principle Predicting Complexity of Inhibitor Risk: *F8* Genotype in Context of Hla-Ii Heterogeneity

Concurrent with the described *in vitro* work above, clinicians began describing particular missense *F8* genotypes as “high risk” and by implication other genotypes at lower risk. R612C (Human Genome Variation Society (HGVS)-nomenclature) (previously reported as R593C without the 19 amino acid leader sequence), is one such *F8* genotype labeled as “high risk” (27). The INSIGHT cohort demonstrated the strength of an international collaboration to provide a more robust clinical data set to inform individual treaters and patients about risk specific to a given *F8* genotype (21). Analyzing 1,112 non-severe hemophilia A patients from 14 centers performing routine *F8* genotyping (to avoid selection bias), 59 of the 1,112 (5.3%) patients developed an inhibitor. The inhibitor risk at 50 exposure days was 6.7% and at 100 exposure days rising to 13.3%. Of the total 214 different *F8* genotypes described in that study, 19 were associated with a detectable inhibitor, provoking more questions than answers. For the 19 “at risk” *F8* genotypes with reported inhibitors, what were the determinants of risk for an inhibitor to occur and for the majority of genotypes without reported inhibitors, could it really be concluded that they were at meaningfully different risk to those in the inhibitor positive *n* = 19 subgroup? Some environmental risk factors have been identified (e.g., treatment intensity, peak level of treatment), but the question remained at the larger cohort level what might the determinants be for a given *F8* genotype that could predict inhibitor risk? (28) Could the simplicity of the single amino acid difference between t-FVIIII and e-FVIII within the complex, multi-domain FVIII protein of 2,332 amino acids be re-evaluated as a function of HLA-FVIII peptide presentation?

**Figure 1:**
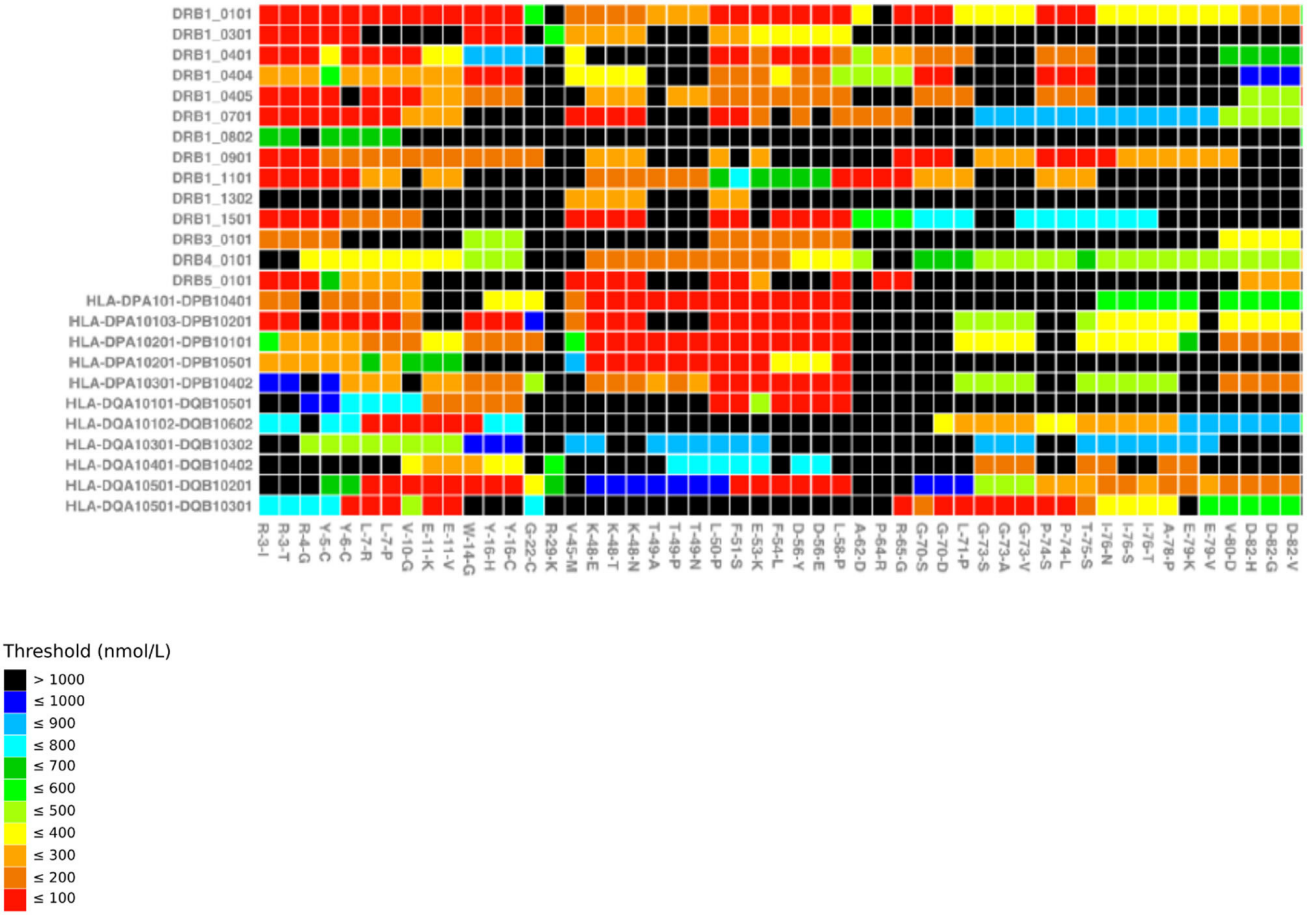
MHC-binding strengths of *F8* peptides predicted to form novel pMHC surfaces. Heatmap showing the predicted occurrence of novel pMHC surfaces and binding strengths for 26 HLA-DR/DP/DQ alleles (*y* axis) covering the first 51 missense mutations in f8-db-eahad.org database (*x* axis). Black squares indicate *F8* missense mutation/HLA allele combinations that are not predicted to form a novel pMHC surface. Otherwise the temperature color scale indicates the predicted binding strength of the strongest binding peptide with a novel pMHC surface for each remaining *F8* missense mutation/HLA allele combination (31).

Shepherd et al. published a large scale *in silico* study to demonstrate the predicted importance of interpreting *F8* genotype in the context of HLA-DR type and the inherent heterogeneity in this. Utilizing a well-established *in silico* class II MHC peptide binding prediction server (NetMHCII), they modeled 520 *F8* missense genotypes (at 392 locations within the *F8* gene) through 14 common HLA-DR types (with 70% population coverage) comparing endogenous vs. therapeutic FVIII-derived 15 mer amino acid sequences straddling the causative *F8*-mutation position (29, 30). The authors make explicit the calculated scale up of HLA-DR/15 mer peptide combinations required for this, with 5,880 different tFVIII-derived peptide possibilities and 7,280 endogenous FVIII-derived peptides, each modeled through the panel of 14 HLA-DR isoforms. This resulted in 1,340,640 separate calculations. The resulting published heat maps (Figure 1) of predicted strongest binding candidate peptide for each *F8* genotype and HLA-DR combination visually depicts the heterogeneity of inhibitor risk prediction, not solely dependent on *F8* genotype alone for the majority. Interestingly, for a minority of *F8* genotypes, regardless of the HLA-DR isoform, a novel peptide-MHC surface could be generated with the potential to provoke a Th cell response, including the aforementioned R593C. Such apparent promiscuity for any HLA-DR type in the panel was evident for 15 of the *F8* genotypes (K166E, K166T, F293S, T295A, T295I, A469G, A469T, A469V, R593C, M614I, F1775P, A1779P, R2150C, R2150H, H2155D).

Recognizing that all patients with identical *F8* mutations are not at the same risk of inhibitor formation, but in the absence of routinely available HLA typing data required for the majority of genotypes in the Shepherd model (29), Pashov et al. published a pragmatic, weighted *F8* genotypic risk stratification (32). Authors derived the mean predicted peptide binding strength *in silico* for an HLA-DR panel using the “Immune Epitope Database” (IEDB). This platform incorporates 5 different predictive *in silico* platforms into a single, consensus meta-algorithm. The calculated mean affinity of t-FVIII-derived peptides for 10 HLA Class II alleles assigned each *F8* genotype a “promiscuity index” ranging from 0 to 100, zero being consensus predicted high affinity binder, to 100, low binding affinity. Inhibitor positive cases demonstrated a significantly more promiscuous peptide affinity prediction than the inhibitor negative cases derived from the EAHAD registry. Both Shepherd and Pashov et al. authors make the case for scaled up, *in silico* prediction servers at such a scale that could not be feasible *in vitro* to expand our insight into the complexity of HLA antigen presentation of each individual missense *F8* mutation (29, 32). Importantly, the predictive power of the utilized algorithms had been previously validated against real peptide binding data (not used to teach each algorithm). It should be remembered that a peptide predicted to bind a given HLA allele with high affinity does not guarantee T cell activation. Shepherd et al. recognized that their predictive algorithm “overcalled” risk, relative to the reported prevalence of clinically detectable inhibitor responses (29). This could be explained by clinical surveillance practice, threshold sensitivity of the clinical assays or insufficient cumulative or intensity of FVIII treatment to some patients (21, 23, 33). However, subsequent studies, below, elaborate on additional antigen presentation or tolerance mechanisms that may reduce further the risk of inhibitor predicted from peptide binding affinity alone.

Kempton and Payne's clinical cohort study contributes confirmatory, individualized clinical data to how *in silico* FVIII-derived peptide binding predictions furthers our understanding of an apparent threshold of activation for inhibitor development (34). In contrast to the Shepherd and Pashov papers that used the EAHAD *F8* repository (29, 32), genotype-specific inhibitor rates in the absence of patient level HLA typing, Kempton and Payne describe a smaller patient cohort (*n* = 57), but each individually HLA typed and correlated with inhibitor status (34). Twenty inhibitor positive cases and 37 inhibitor negative controls had predictions of peptide binding and subsequent upward, T cell receptor (TCR) facing, novel peptide-MHC surface as described by Shepherd et al. (29). The t-FVIII derived peptide was considered novel if it was predicted to bind, present to a TCR and be unique from that presented by the e-FVIII derived peptides. Candidate peptide binding predictions used the www.iedb.org server. Authors found the prediction of binding of FVIII-peptides to a patient's own HLA-DRB1, creating a novel peptide-MHC surface to interact with the TCR, was strongly associated with inhibitor development as predicted by Shepherd et al. (29). Additionally, aligning with Pashov's predictions (32), there is an apparent burden of novel peptide presentation that is required to provoke a clinically detectable inhibitor. Kempton applied a predicted >10 novel peptide-MHC surfaces per patient to be a meaningful threshold resulting in an overall risk (OR) increase of 4.4 (95% CI 1.1–15.0), adjusted for intensive FVIII treatment (34). Additionally, their data suggests higher levels of HLA-DRB1 binding and resultant novel pMHC surfaces for some *F8* genotypes identified previously as “higher risk” (e.g., R593C). Although patient numbers are small and larger clinical cohorts would be required to confirm this, the threshold effect of multiple peptides rather than a single peptide available to drive the adaptive immunological response is compelling.

Although the studies discussed thus far have derived statistical significance in their prediction of inhibitor risk, there remains a concern that computational predictions continue to overcall risk. Hart et al. put forward a novel hypothesis of coincidental and previously unrecognized tolerance to tFVIII-derived peptides attributable to predicted cross matched primary peptide sequence homology between tFVIII and unrelated proteins in the human proteome as a possible explanation of this over calling (31). This emanated from an initial hypothesis that the known structural and sequence homology between FVIII and FV might afford some coincidental primary peptide sequence homology, providing additional central tolerance to t-FVIII-derived peptides. They extend their predictions to 25 common HLA DR, DP, DQ isoforms with estimated worldwide population coverage of >70, >90, and >80%, respectively and 956 distinct *F8* missense mutations at 605 different loci from 3,243 individuals, a total of 160 (4.9%) of whom were identified as having an inhibitor. The experimental design is based on their previous work described by Shepherd et al. and also Kempton and Payne (29, 34), identifying HLA-II binding, t-FVIII-derived peptides that form a predicted upward-facing, novel p-MHC surface to interact with helper TCRs. Layered on top of this extended repertoire of HLA and *F8* genotypes is a comprehensive cross referencing of all putative FVIII-derived HLA-II core binding 9 mer peptide sequences with the primary sequences of the 20,000 proteins constituting our human proteome (www.uniprot.org/). After subdivision into all possible 9 mers, the canonical human proteome consists of 39 million 9-mers, 11 million of which are non-identical. The predicted novel FVIII-p-MHC surfaces from previous work are cross referenced against this human proteome 9 mer repository and are required to remain unique to still be reclassified as a novel p-MHC surface capable of stimulating an engaged Th cell. Four thousand six hundred and five proteins of the 20,300 within the human proteome afforded some cross matching. Factor V afforded the most cross-matching, then Hephestin-like protein 1 and Ceruluplasmin with 640, 457, and 437 homologous protective peptides, respectively. The consequent predicted cohort-wide inhibitor risk falls appreciably from 37 to 21% with a binding threshold of 500 nM. Validating *in vitro* experiments are still required to demonstrate tolerance to proteome cross-matched peptides in contrast to those binding peptides without a proteome cross-match. Specifically, *in vitro* demonstration of T cell reactivity to relevant peptides/HLAII combinations without predicted proteome crossmatching, and the absence of equivalent reactivity to proteome-cross matched peptides/HLAII straddling the same *F8* mutation position, will be an important *in vitro* validation of this hypothesis.

Hart et al. acknowledge that potential confounders remain, limiting the repertoire of FVIII-derived peptides available for MHC presentation (31). Addressing these, Schneidman-Duhovny et al. provide a step-change refinement in their *in silico* pipeline to further improve prediction accuracy (35). Specifically, a three step, “integrative structure-based” algorithm starts with a peptide cleavage prediction to account for the cleavage preferences of natural intracellular proteases, cathepsins B, H, and S (36, 37). The second step not only used the “conventional” peptide binding prediction servers already described, but further trained the output from IEDB with an atomic distance-dependent statistical potential to better account for stability of the predicted peptide MHC interaction (38). Finally, previously described modeling was only of peptide-MHC surface available to interact with a given TCR without any account of the variable footprints TCRs might take over a given p-MHC surface. This new pipeline incorporates a structure-based predictor of peptide MHC-II—TCR recognition. Their data includes a validating peptide series, unrelated to FVIII, but subsequently use FVIII derived peptides as a proof of translational principle, in particular to narrow the field of likely preferred tFVIII-derived binders. Using 5 patient-derived TCR sequences reduced the number of possible 12 mer epitope cores from 2,340 to just six peptides including the correct epitope core (35). Such refinement is hypothesis generating, providing a manageable repertoire of candidate immunogenic peptides with which to work.

Finally, van Haren et al. provide important data highlighting the cellular context of antigen presentation (39). Maturity status of dendritic cells processing FVIII-derived peptides correlated with the efficiency of membrane presentation of peptide loaded HLA Class II, less mature DCs retaining more of the peptide-loaded HLA molecules intracellularly. Additionally, macrophages were also able to take up and process FVIII, albeit less efficiently than DCs. Van Haren concludes a relatively limited number of FVIII peptides are presented by multiple HLA-DR molecules, although this experimental technique may preferentially detect immunodominant peptides. Additional work documents the contribution of HLA-DQ to antigen presentation (40).

van Haren observes only a minority of the peptides predicted *in silico* to bind, actually bind to HLA class II and are retrievable in peptide elution experiments (40). The Schneidman cathepsin-cleaving modeling is likely to contribute to this more limited repertoire (35). Competition from the multitude of other intracellular self and non-self peptides vying for presentation position within the HLAII repertoire has not been accounted for, although may be an explanation, in part, for the limited retrievable repertoire. Given the Kempton proposed requirement of multiple presented FVIII-derived peptides to drive a clinically relevant response (34), the van Haren data demonstrating a more limited repertoire of actually available peptides contributes further to explain why clinically observed inhibitor responses are lower than might be predicted (40), and goes some way to answering the question, not “*why has this individual generated an antibody response*,” but rather “*why has this individual not generated an anti-FVIII antibody response?*”

This series of clinical epidemiology, *in vitro* and *in silico* data sets have, together, highlighted the complexity of antigen presentation at the time of an exogenously infused protein therapeutic, such as FVIII. The computational power of *in silico* algorithms has been an absolute necessity to re-evaluate the predicted importance of HLA haplotypes to inhibitor risk in our non-severe hemophilia A patient cohorts, but also the limitation of simplifying risk stratification to just the *F8* genotype and HLA class II combination. Future evolution and sophistication of immunological predictive pipelines, incorporating additional steps within the antigen processing pathway as alluded to in this review, will further elucidate mechanism of the allo-response against therapeutic FVIII protein and refine personalized inhibitor prediction accuracy. The whole-genome sequencing era opens the opportunity for a renewed, coordinated and systematic effort between clinical and laboratory teams to further characterize their patients' profiles sufficiently to contribute the necessary, validatory, real-world data for these pipelines. This will be the crucial step to achieve meaningful translation of this technology for patient benefit.

## Author Contributions

The author confirms being the sole contributor of this work and has approved it for publication.

## Conflict of Interest

The author declares that the research was conducted in the absence of any commercial or financial relationships that could be construed as a potential conflict of interest.
